# Supplementary material for: Basal ganglia components have distinct computational roles in decision-making dynamics under conflict and uncertainty
Source: PLoS Biol. 2025 Jan 23;23(1):e3002978. doi: 10.1371/journal.pbio.3002978 (PMC11756759; doi:10.1371/journal.pbio.3002978)
Supplement: S1 Text — (DOCX) [file pbio.3002978.s001.docx]

## 1. Additional Study Information

Patients used a joystick (Penny & Giles JC150) to guide a dot on the screen towards a left or right response field, indicating the primary direction of moving dots. Response times and actions were precisely recorded using 'MonkeyLogic' in Matlab (49), a software tool that yields temporally precise and comprehensive behavioral data for systems neuroscience experiments. In the version for students, participants used the left or right index finger on the triggers of a Logitech F310 game controller, with responses recorded by Psychtoolbox in Matlab.

DBS targeting was performed using a combination of indirect (AC-PC coordinate system), direct (MRI target visualization) and neurophysiological methods. Specifically, neuroimaging was used to design the initial trajectories to target, and electrophysiology was then monitored intra-operatively to identify the best trajectory for lead implantation in each hemisphere. We identified particular brain regions (GPi, GPe, and STN) based on their unique firing properties, recorded with Alpha Omega (“AO,” Nazareth, ISRAEL) microphonic-free microelectrodes and monitored via the AO NeuroOmega system. This process was overseen by an experienced neurophysiologist (WFA). Task-recordings were undertaken when single-unit activity was consistent with electrode proximity to target regions and with patient consent. Post-operative imaging, augmented with atlas co-registration, was employed to confirm final lead position and to reconstruct microelectrode recording sites along the trajectories. This multi-modal approach, along with intra-operative DBS stimulation testing, ensured accurate targeting within the brain's subcortical structures.

To precisely determine the anatomical source of neural signals, we employed a multimodal strategy combining physiological signal interpretation and post-operative imaging. The differentiation between the GPi and the GPe was based on their distinct firing properties. During electrode insertion, it was often possible to identify the lamina separating these structures by observing changes in firing patterns. This process involved continuous monitoring of the electrode’s trajectory and recording neuronal activity at various depths. The STN was distinctly identified by its unique physiological characteristics, which are markedly different from adjacent structures. Signal interpretation was undertaken by experts possessing extensive experience in human and nonhuman primate neurophysiological analysis. Experimental recordings were conducted when visual and auditory analyses of single-unit activity indicated that two or more trajectories intersected a region of interest. Additionally, patient willingness to participate in the task was a prerequisite. Furthermore, post-operative imaging (MRI with leads in-place) was combined with atlas co-registration to precisely ascertain lead placements and then to accurately reconstruct the recording tracts (the final lead depth is precisely known relative to intra-op recording sites). This integrative approach maximized confidence regarding the source of neural signals used in this study.
