# Supplementary material for: Basal ganglia components have distinct computational roles in decision-making dynamics under conflict and uncertainty
Source: PLoS Biol. 2025 Jan 23;23(1):e3002978. doi: 10.1371/journal.pbio.3002978 (PMC11756759; doi:10.1371/journal.pbio.3002978)
Supplement: S1 Fig — (DOCX) [file pbio.3002978.s002.docx]

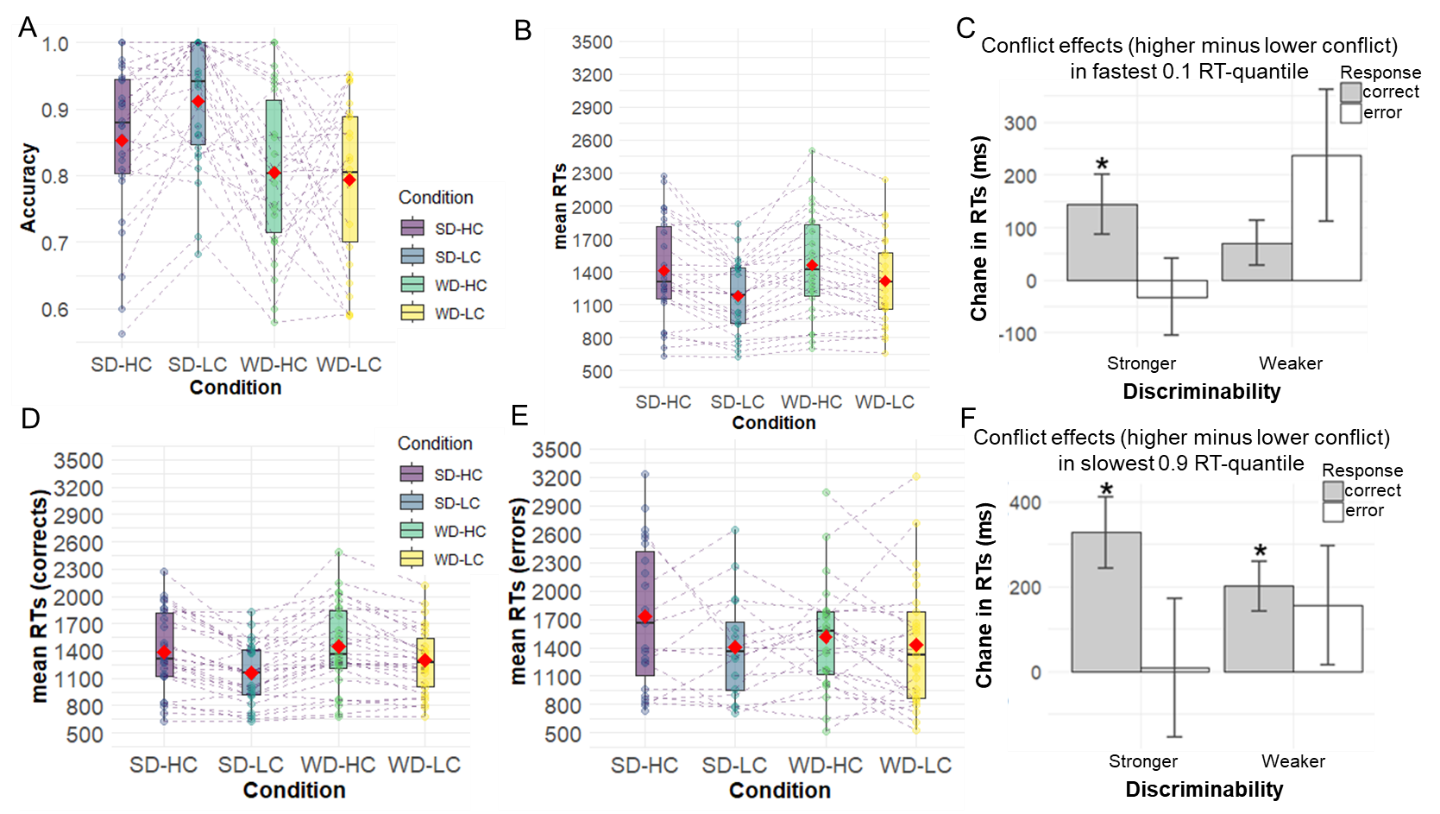
S1 Fig. Summary statistics of accuracy, mean response times, & conflict effects.

**(A)** Distribution of accuracy across the four conditions (SD-LC=stronger discriminability, lower conflict; SD-HC=stronger discriminability, higher conflict; WD-LC=weaker discriminability, lower conflict; WD-HC=weaker discriminability, higher conflict). Each condition is represented by a boxplot summarizing the data distribution, overlaid with individual data points for each subject (small circles), and lines connecting these points across conditions to illustrate individual trends. The means for each condition are highlighted by large red diamonds. **(B)** Distribution of mean response times (RTs), averaged over error and correct responses, across the four conditions. Each condition is represented by a boxplot summarizing the data distribution, overlaid with individual data points for each subject (small circles), and lines connecting these points across conditions to illustrate individual trends. The means for each condition are highlighted by large red diamonds. **(C)** Changes in the 10% fastest reaction times (i.e., 0.1 RT quantile) for high minus low conflict trials. Asterisks indicate significance (p<0.05; Wilcoxon signed-rank tests for paired samples: Test statistics for conflict difference under stronger discriminability: V = 281, N = 26, p = 0.006, r = -0.537). RT quantiles were calculated by subject and then averaged across subjects. **(D)** Distribution of mean response times for correct responses across the four conditions. Each condition is represented by a boxplot summarizing the data distribution, overlaid with individual data points for each subject (small circles), and lines connecting these points across conditions to illustrate individual trends. The means for each condition are highlighted by large red diamonds. **(E)** Distribution of mean response times for error responses across the four conditions. Each condition is represented by a boxplot summarizing the data distribution, overlaid with individual data points for each subject (small circles), and lines connecting these points across conditions to illustrate individual trends (note that individuals with accuracy at 100% do not have lines for these conditions). The means for each condition are highlighted by large red diamonds. **(F)** Changes in the 90% slowest reaction times (i.e., 0.9 RT quantile) for high minus low conflict trials. Asterisks indicate significance (p<0.05; Wilcoxon signed-rank tests for paired samples. Test statistics for conflict difference under stronger discriminability: V = 309, N = 26, p < 0.001, r = -0.706. Test statistics for conflict difference under weaker discriminability: V = 288, N = 26, p = 0.003, r = -0.577). RT quantiles were calculated by subject and then averaged across subjects. We provide data and corresponding analyses scripts for reproducing figures on:

<https://osf.io/k38pj/?view_only=5c442294fcfb4991bb42cd902c60249c>
