# Supplementary material for: Basal ganglia components have distinct computational roles in decision-making dynamics under conflict and uncertainty
Source: PLoS Biol. 2025 Jan 23;23(1):e3002978. doi: 10.1371/journal.pbio.3002978 (PMC11756759; doi:10.1371/journal.pbio.3002978)
Supplement: S2 Fig — (DOCX) [file pbio.3002978.s003.docx]

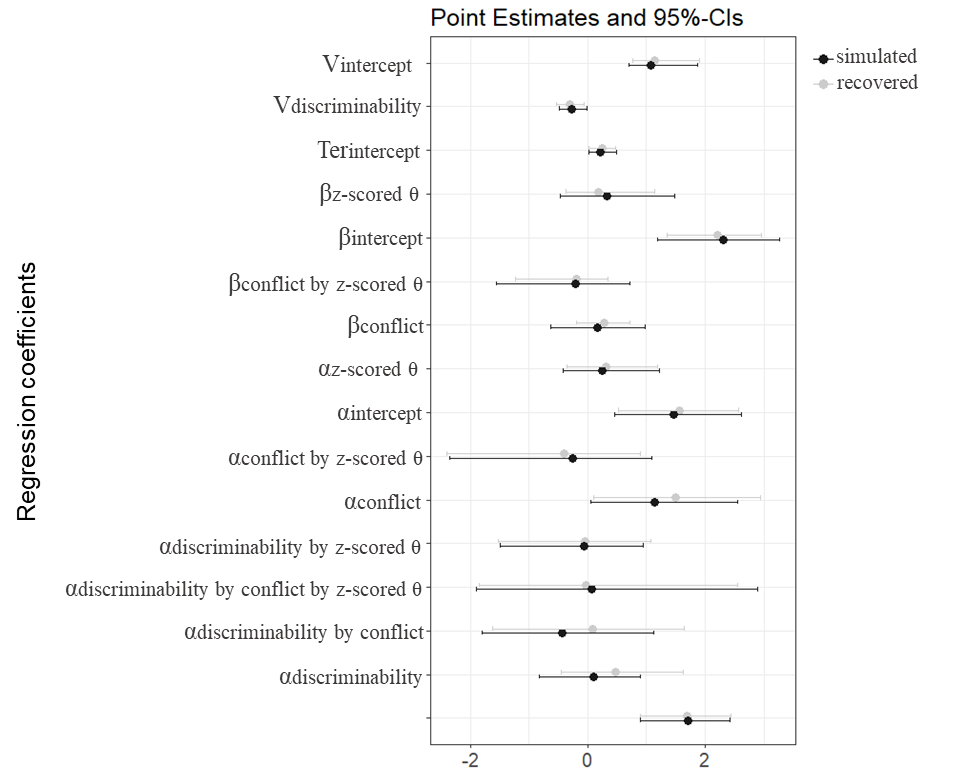


S2 Fig. Results from parameter recovery analyses of best-fitting model.

Results from simulations comparing inputted parameters (black) and recovered parameters (grey). Overall, these plots show good parameter recovery. Shown are means (dots) and corresponding 95% highest density interval. The parameter labels refers to the regression equations in the Methods. We provide data and corresponding analyses scripts for reproducing figures on:

<https://osf.io/k38pj/?view_only=5c442294fcfb4991bb42cd902c60249c>
