# Supplementary material for: Basal ganglia components have distinct computational roles in decision-making dynamics under conflict and uncertainty
Source: PLoS Biol. 2025 Jan 23;23(1):e3002978. doi: 10.1371/journal.pbio.3002978 (PMC11756759; doi:10.1371/journal.pbio.3002978)
Supplement: S3 Fig — (DOCX) [file pbio.3002978.s004.docx]

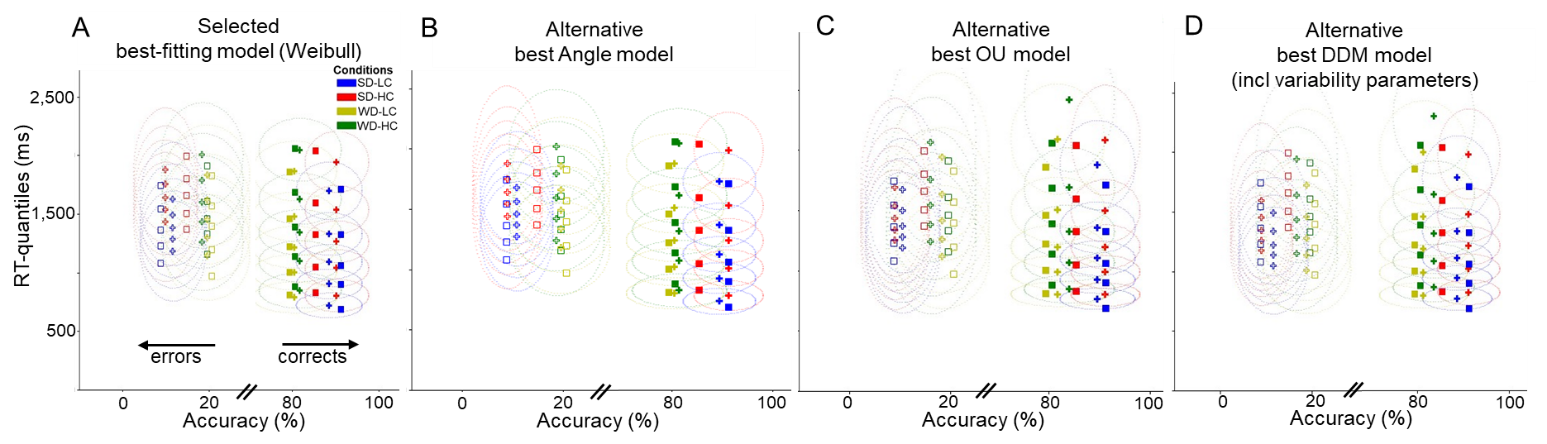
S3 Fig. Posterior predictive checks of best-fitting model and alternative models.

Posterior predictive check of best-fitting model. Squares indicate data; crosses indicate posterior predictions. Ellipses surrounding crosses indicate 95% confidence intervals in expected range of data given stochasticity in model and estimation uncertainty. All measures were calculated by condition and by subject before averaging. The models are specified in Supplementary Table 2. **A.** best-fitting model that was selected for the main analyses discussed in the main manuscript (Supplementary Table 2: row 1). **B**. best model in the category “Angle” (i.e., models with a linear collapse), labeled as model 5 in the Supplementary Table 2. **C.** best model in the category “OU” (i.e., models with an Ornstein-Uhlenbeck evidence accumulation process), labeled as model 10 in the Supplementary Table 2. **D.** best model in the category “DDM” (i.e., classical diffusion decision model with all variability parameters), labeled as model 14 in the Supplementary Table 2. We see systematic misfits such that the best DDM and OU models systematically misfit the tails of particularly correct responses. The best Angle model systematically misfits the leading edge of errors in the SD-LC condition as well as accuracy levels in the SD-HC condition. We provide data and scripts on:

<https://osf.io/k38pj/?view_only=5c442294fcfb4991bb42cd902c60249c>
