# Supplementary material for: Basal ganglia components have distinct computational roles in decision-making dynamics under conflict and uncertainty
Source: PLoS Biol. 2025 Jan 23;23(1):e3002978. doi: 10.1371/journal.pbio.3002978 (PMC11756759; doi:10.1371/journal.pbio.3002978)
Supplement: S4 Fig — (DOCX) [file pbio.3002978.s005.docx]

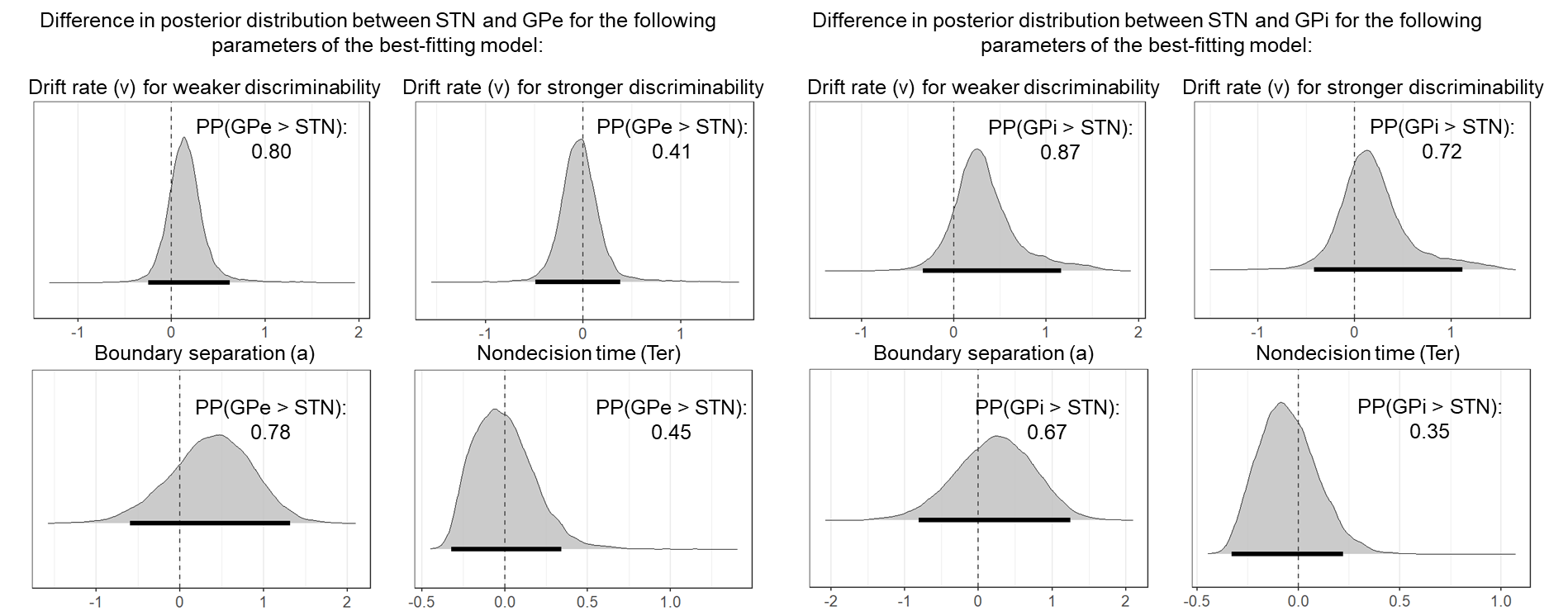


S4 Fig. Difference in posterior distributions of best-fitting model parameters between STN and GPe and STN and GPi.

Comparisons of model parameters governing collapse onset and shape are shown in the main manuscript. The posterior estimates (including credible intervals) for each group are shown in the Supplementary Table 3. We provide data and scripts on:

<https://osf.io/k38pj/?view_only=5c442294fcfb4991bb42cd902c60249c>
