# Supplementary material for: Basal ganglia components have distinct computational roles in decision-making dynamics under conflict and uncertainty
Source: PLoS Biol. 2025 Jan 23;23(1):e3002978. doi: 10.1371/journal.pbio.3002978 (PMC11756759; doi:10.1371/journal.pbio.3002978)
Supplement: S5 Fig — (DOCX) [file pbio.3002978.s006.docx]

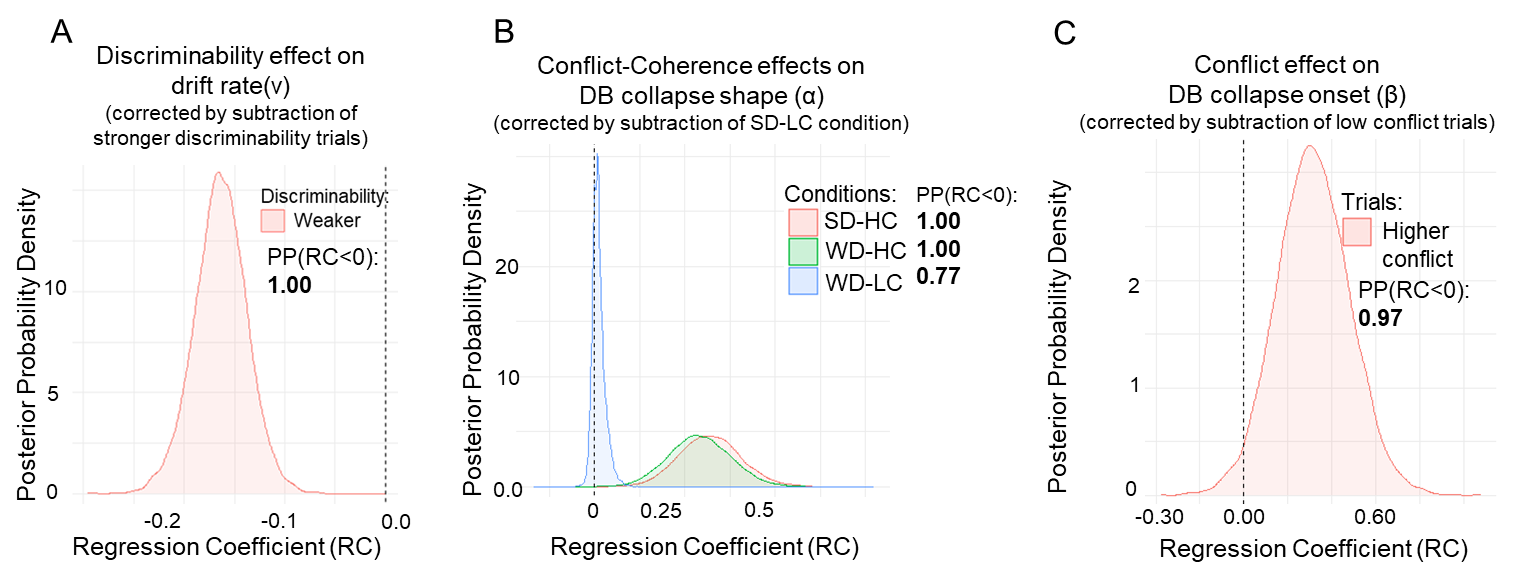


S5 Fig. Posterior estimates of best-fitting (behavioral) Weibull-DDM for the student sample without neurological conditions.

**(A)** Main effect of stronger-weaker discriminability on drift rate. Shown is the difference in posterior distribution of drift rates (v) between weaker versus stronger discriminability trials. Posterior probability (PP): v_stronger_ > v_weaker_ = 1.00. **(B)** Conflict-by-coherence interaction, leading to more concave boundary collapse for high conflict particularly under stronger coherence (SD-HC). Shown are differences in posterior distribution of collapse shapes (α) of conditions relative to the easiest SD-LC condition. Posterior probability (PP): α_SD-HC_ > α_WD-LC_ = 1.00; α_WD-HC_ < α_WD-LC_ = 1.00; α_WD-HC_ > α_SD-HC_ = 0.74. **(C)** Main effect of high-low conflict on the onset of boundary collapse. Shown is the difference in posterior distribution of collapse onsets (β) between higher versus lower conflict trials. Posterior probability (PP): β_higher_ > β_lower_ = 0.97. We provide data and scripts on:

<https://osf.io/k38pj/?view_only=5c442294fcfb4991bb42cd902c60249c>
