# Supplementary material for: Basal ganglia components have distinct computational roles in decision-making dynamics under conflict and uncertainty
Source: PLoS Biol. 2025 Jan 23;23(1):e3002978. doi: 10.1371/journal.pbio.3002978 (PMC11756759; doi:10.1371/journal.pbio.3002978)
Supplement: S6 Fig — (DOCX) [file pbio.3002978.s007.docx]

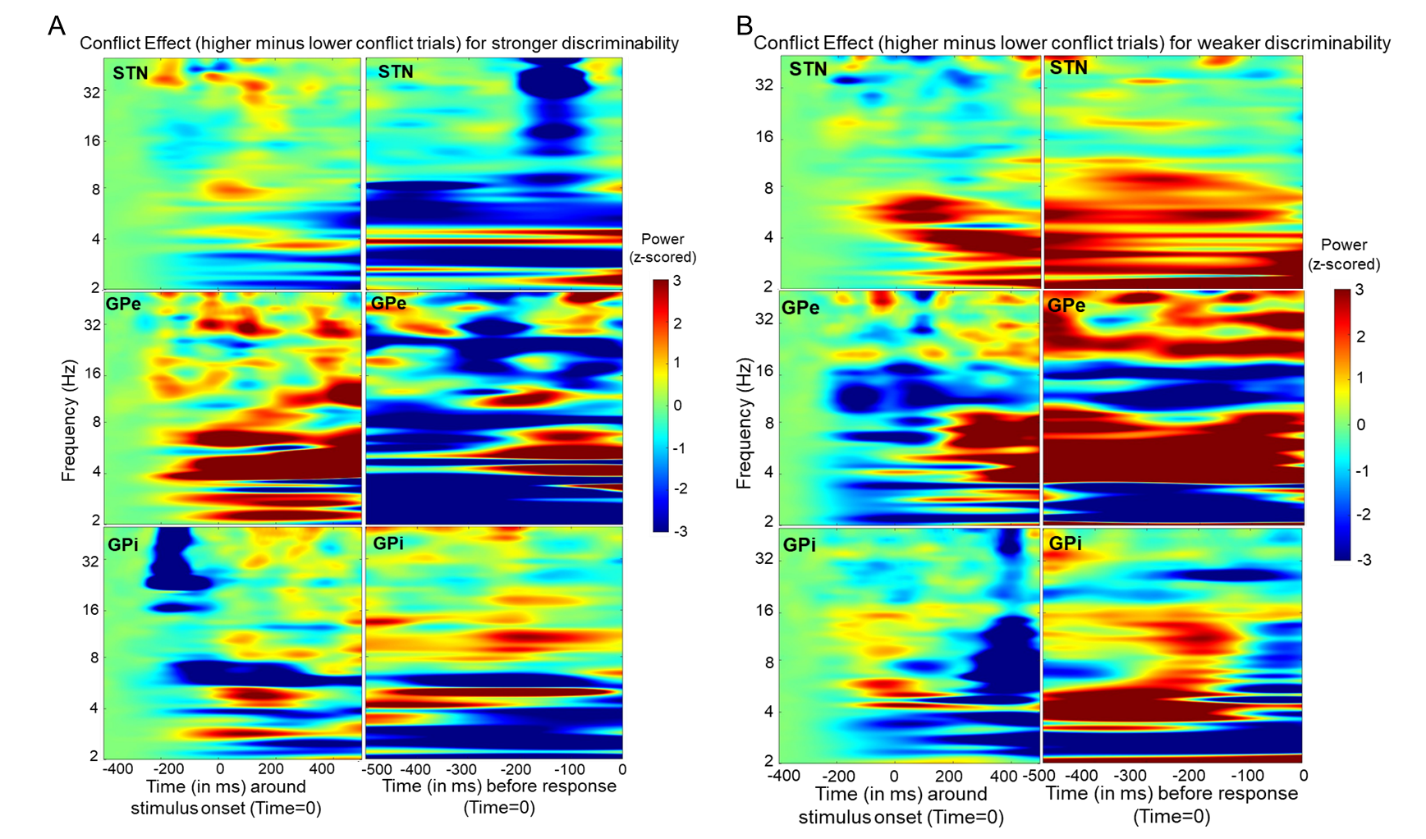


S6 Fig. Task-related neuronal response by discriminability condition.

**(A)** Time frequency plots show a task-related increase in LFO power (averaged across channels) relative to baseline. Spectra are shown for high minus low conflict for stronger discriminability aligned to stimulus onset (left panel) and response (right panel) for each BG component. **(B)** Time frequency plots show a task-related increase in LFO power (averaged across channels) relative to baseline. Spectra are shown for high minus low conflict for weaker discriminability aligned to stimulus onset (left panel) and response (right panel) for each BG component. We provide scripts on:

<https://osf.io/k38pj/?view_only=5c442294fcfb4991bb42cd902c60249c>
