# Supplementary material for: Basal ganglia components have distinct computational roles in decision-making dynamics under conflict and uncertainty
Source: PLoS Biol. 2025 Jan 23;23(1):e3002978. doi: 10.1371/journal.pbio.3002978 (PMC11756759; doi:10.1371/journal.pbio.3002978)
Supplement: S7 Fig — (DOCX) [file pbio.3002978.s008.docx]

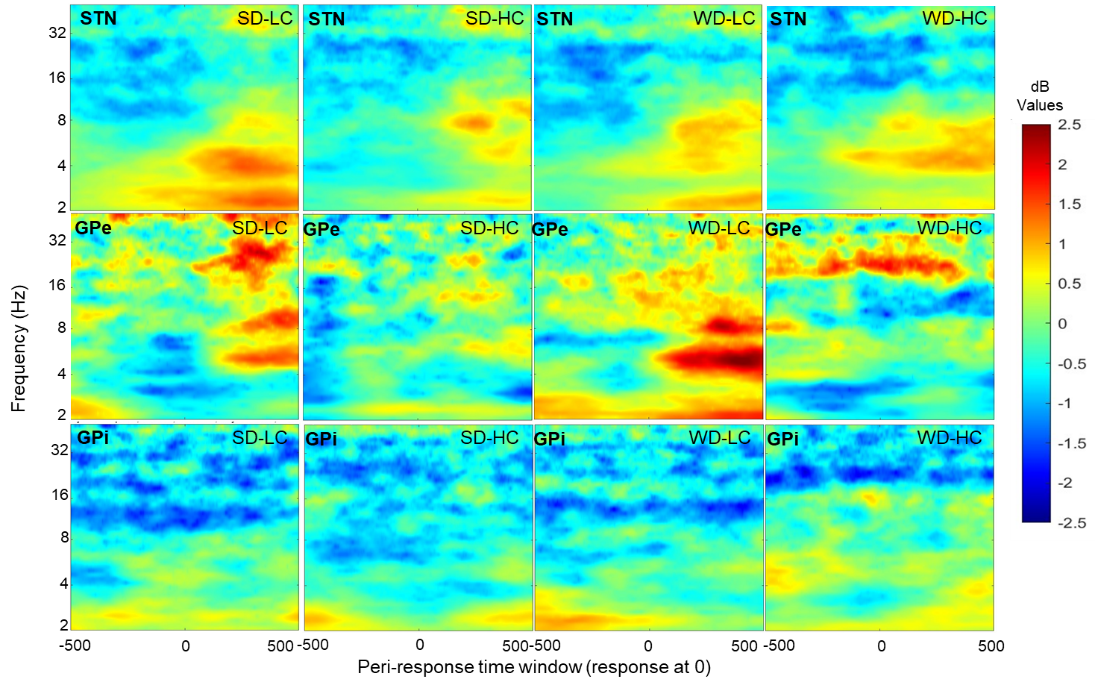


S7 Fig. Peri-response activity by task conditions.

Task-related increase in dB power (averaged across channels) relative to baseline for each task condition and BG component. Right top refers to task condition (SD-HC = stronger discriminability, higher conflict; SD-LC = stronger discriminability, lower conflict; WD-LC = weaker discriminability, lower conflict; WD-HC = weaker discriminability, higher conflict. This data pattern shows beta desynchronization that is most evident in the STN, with decreasing prominence in the GPi and GPe. It is important to note that the use of larger electrodes (macro LFPs), as opposed to our micro-LFPs, might have likely accentuated this phenomenon. This is because beta desynchronization typically reflects disruptions in broader spatial correlations within the beta band, rather than merely a localized reduction in beta power (although the latter might also occur but probably more heterogeneously across different substructures). We provide scripts on:

<https://osf.io/k38pj/?view_only=5c442294fcfb4991bb42cd902c60249c>
