# Supplementary material for: Basal ganglia components have distinct computational roles in decision-making dynamics under conflict and uncertainty
Source: PLoS Biol. 2025 Jan 23;23(1):e3002978. doi: 10.1371/journal.pbio.3002978 (PMC11756759; doi:10.1371/journal.pbio.3002978)
Supplement: S8 Fig — (DOCX) [file pbio.3002978.s009.docx]

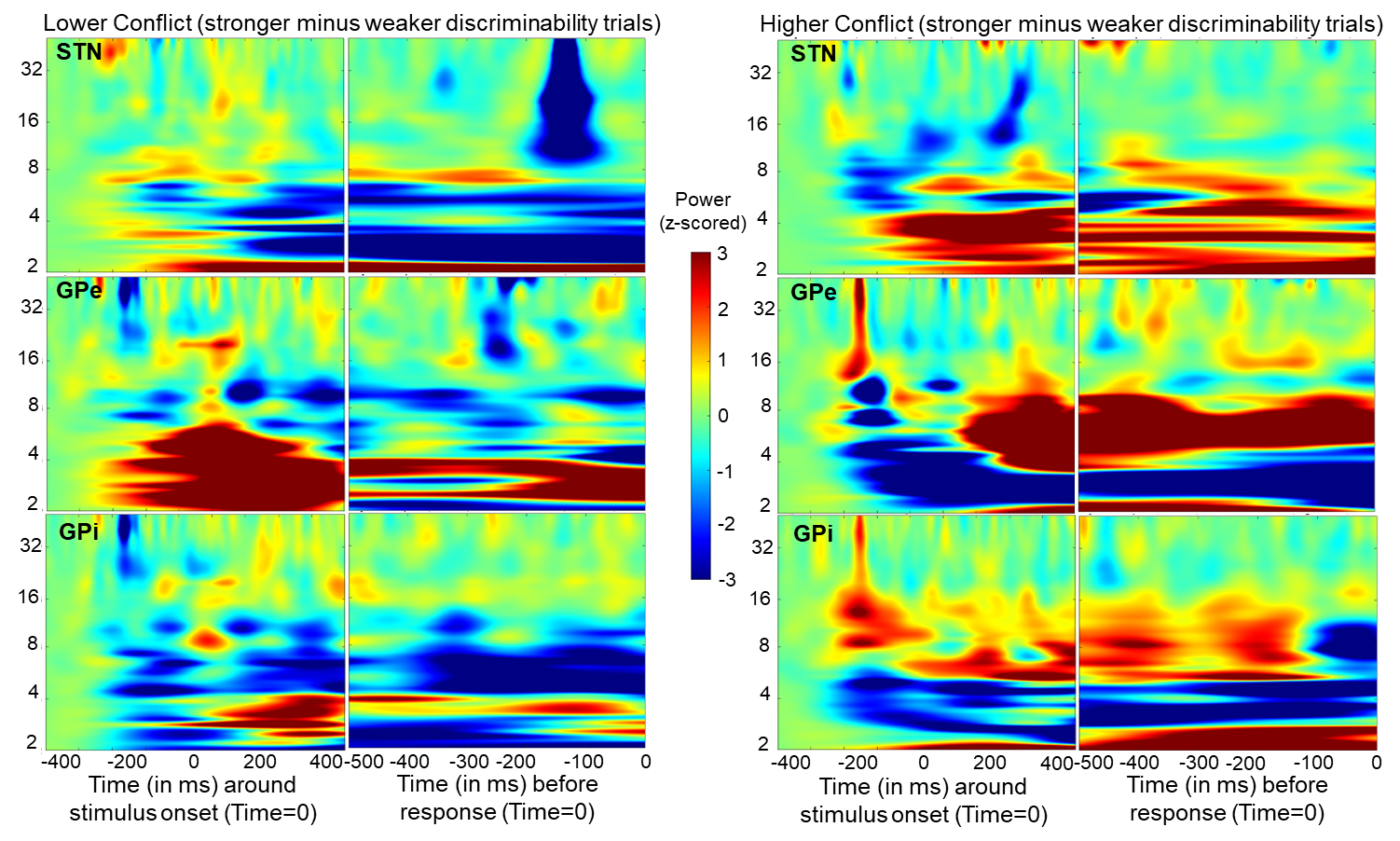
S8 Fig. Task-related neuronal response by conflict condition.

**(A)** Spectra are shown for stronger minus weaker discriminability for lower conflict aligned to stimulus onset (left panel) and response (right panel) for each BG component. **(B)** Spectra are shown for stronger minus weaker discriminability for higher conflict aligned to stimulus onset (left panel) and response (right panel) for each BG component. We provide scripts on:

<https://osf.io/k38pj/?view_only=5c442294fcfb4991bb42cd902c60249c>
