# Supplementary material for: Basal ganglia components have distinct computational roles in decision-making dynamics under conflict and uncertainty
Source: PLoS Biol. 2025 Jan 23;23(1):e3002978. doi: 10.1371/journal.pbio.3002978 (PMC11756759; doi:10.1371/journal.pbio.3002978)
Supplement: S9 Fig — (DOCX) [file pbio.3002978.s010.docx]

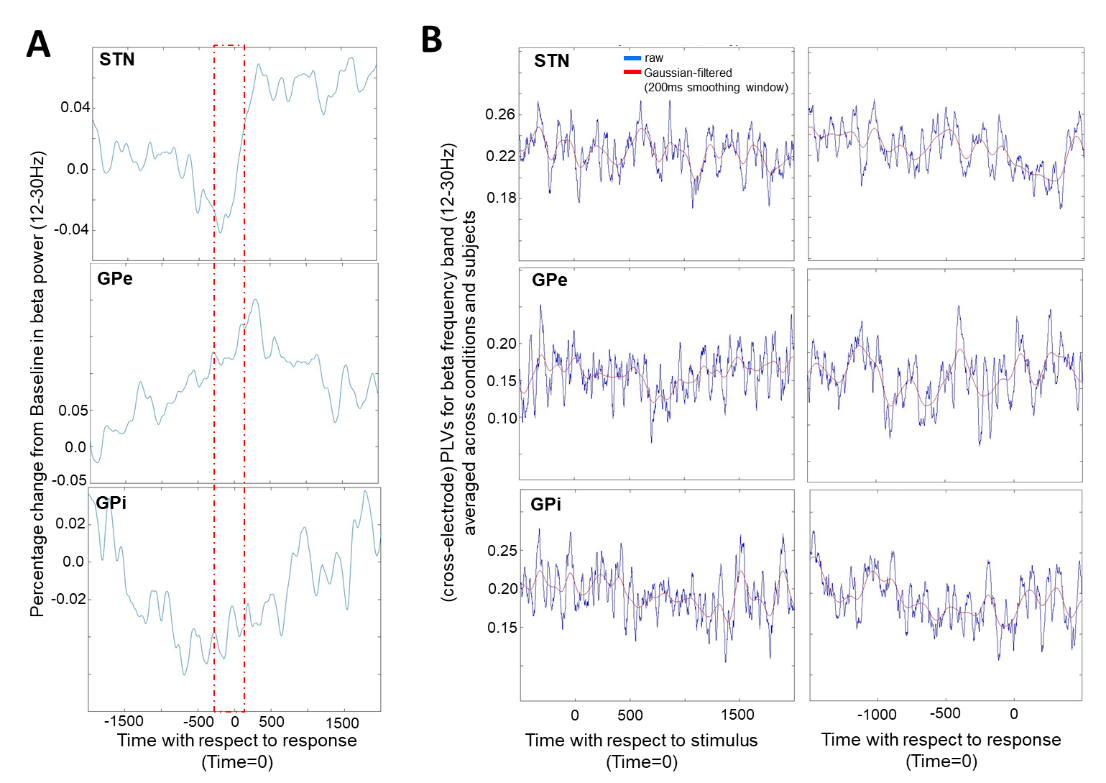


S9 Fig. Results from additional analysis in the beta frequency band.

**(A)** Beta band-filtered change in power across the entire epoch with respect to response. This data pattern (highlighted by the red boxes) shows beta desynchronization that is most evident in the STN, with decreasing prominence in the GPi and GPe. **(B)** Cross-electrode beta-band phase-locking values (PLVs) over time, both in relation to the cue onset (left subplots) and the response (right subplots). This dual perspective underscores the dynamic nature of beta-band correlations across different phases of the task, offering additional insight into the spatial-temporal patterns of neural synchronization. The coherence in PLV patterns across conditions provides evidence that the observed neural activities are linked to cognitive processing rather than noise. Noise-driven signals would display inconsistent PLV patterns, characterized by low or random phase alignment. In contrast, the structured and consistent PLV patterns observed across conditions indicate reliable synchronization of neural activity in response to cognitive demands. To calculate these values, we used the in-built matlab function “pn_eegPLV”. We provide scripts on:

<https://osf.io/k38pj/?view_only=5c442294fcfb4991bb42cd902c60249c>
