# Supplementary material for: Basal ganglia components have distinct computational roles in decision-making dynamics under conflict and uncertainty
Source: PLoS Biol. 2025 Jan 23;23(1):e3002978. doi: 10.1371/journal.pbio.3002978 (PMC11756759; doi:10.1371/journal.pbio.3002978)
Supplement: S10 Fig — (DOCX) [file pbio.3002978.s011.docx]

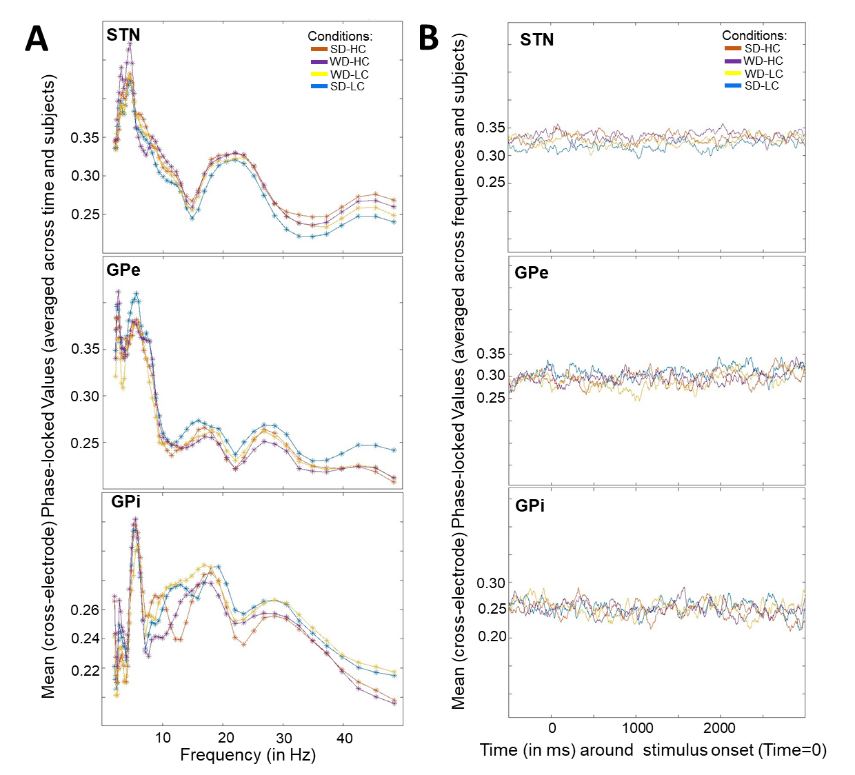


S10 Fig. Results from additional phase-locking values (PLV) analyses.

**(A)**cross-electrode phase-locking values (PLVs) averaged across both time and subjects for each task condition plotted against frequency. PLVs are known as a robust indicator of coordinated brain processes with higher values providing evidence against spurious noise. The PLV patterns show a high degree of similarity across task conditions and frequencies. This consistency underscores that the observed neural activity, as captured by the electrodes, is not an artifact of random noise. Instead, the similar PLV patterns across conditions strongly suggest that the electrodes are detecting common, task-related dynamics. Such uniformity in neural synchrony, irrespective of the experimental condition, reinforces the conclusion that the identified neural signatures are genuinely related to cognition rather than spurious noise. **(B)** PLVs (averaged across all frequencies and subjects) over time and for each condition separately. The high PLVs and the fact that they are similarly fluctuating across the different task conditions over time provides evidence that the neural synchronization captured in the data is not due to random noise but instead suggest common phase patterns between evoked conditions. Moreover, the fact that the fluctuations are not identical but follow a similar trend reflects the underlying neural coherence in response to different task conditions. To calculate these values, we used the in-built matlab function “pn_eegPLV”. We provide scripts on:

<https://osf.io/k38pj/?view_only=5c442294fcfb4991bb42cd902c60249c>
