# Supplementary material for: Basal ganglia components have distinct computational roles in decision-making dynamics under conflict and uncertainty
Source: PLoS Biol. 2025 Jan 23;23(1):e3002978. doi: 10.1371/journal.pbio.3002978 (PMC11756759; doi:10.1371/journal.pbio.3002978)
Supplement: S11 Fig — (DOCX) [file pbio.3002978.s012.docx]

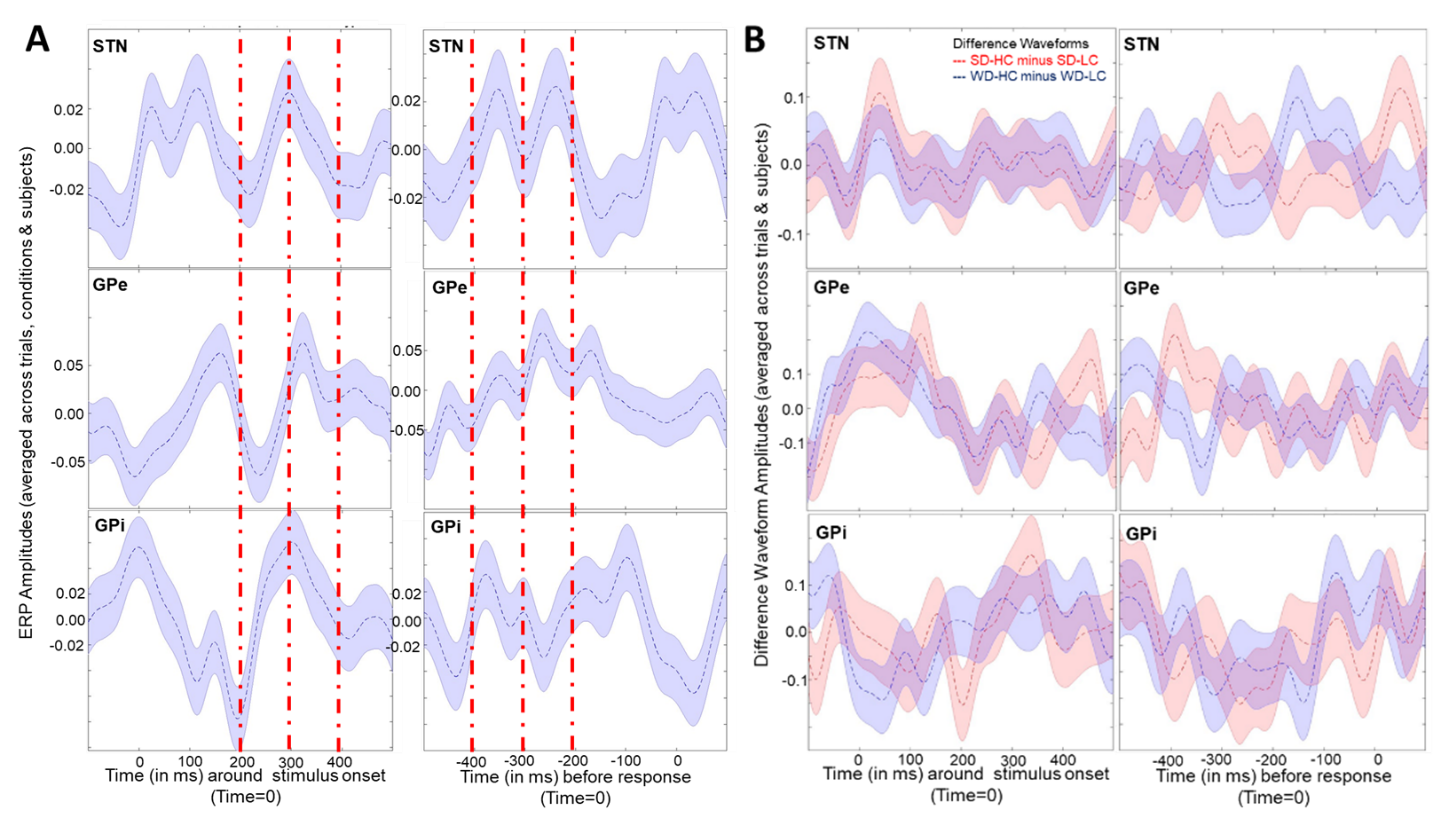


S11 Fig. Results from additional event-related potential (ERP) analyses.

**(A)**grand mean event-related potentials (ERPs) averaged across trials, conditions, and subjects. The blue dotted lines represent the grand mean averages with blue shaded areas referring to the standard errors. This data pattern confirms that the observed phenomena are not merely artifacts confined to specific groups or individuals. The presence of consistent peaks and troughs indicates event-related variations that underlies robust signal-to-noise ratios in our data. **(B)** Difference waveforms demonstrating similarity across conditions, while also distinguishing specific neural components that are selectively activated by different experimental conditions (i.e., higher versus lower conflict for a given discriminability level). This pattern further validates the reliability of our observations and the differential engagement of brain regions in response to distinct task conditions. We provide scripts on:

<https://osf.io/k38pj/?view_only=5c442294fcfb4991bb42cd902c60249c>
