# Supplementary material for: Basal ganglia components have distinct computational roles in decision-making dynamics under conflict and uncertainty
Source: PLoS Biol. 2025 Jan 23;23(1):e3002978. doi: 10.1371/journal.pbio.3002978 (PMC11756759; doi:10.1371/journal.pbio.3002978)
Supplement: S12 Fig — (DOCX) [file pbio.3002978.s013.docx]

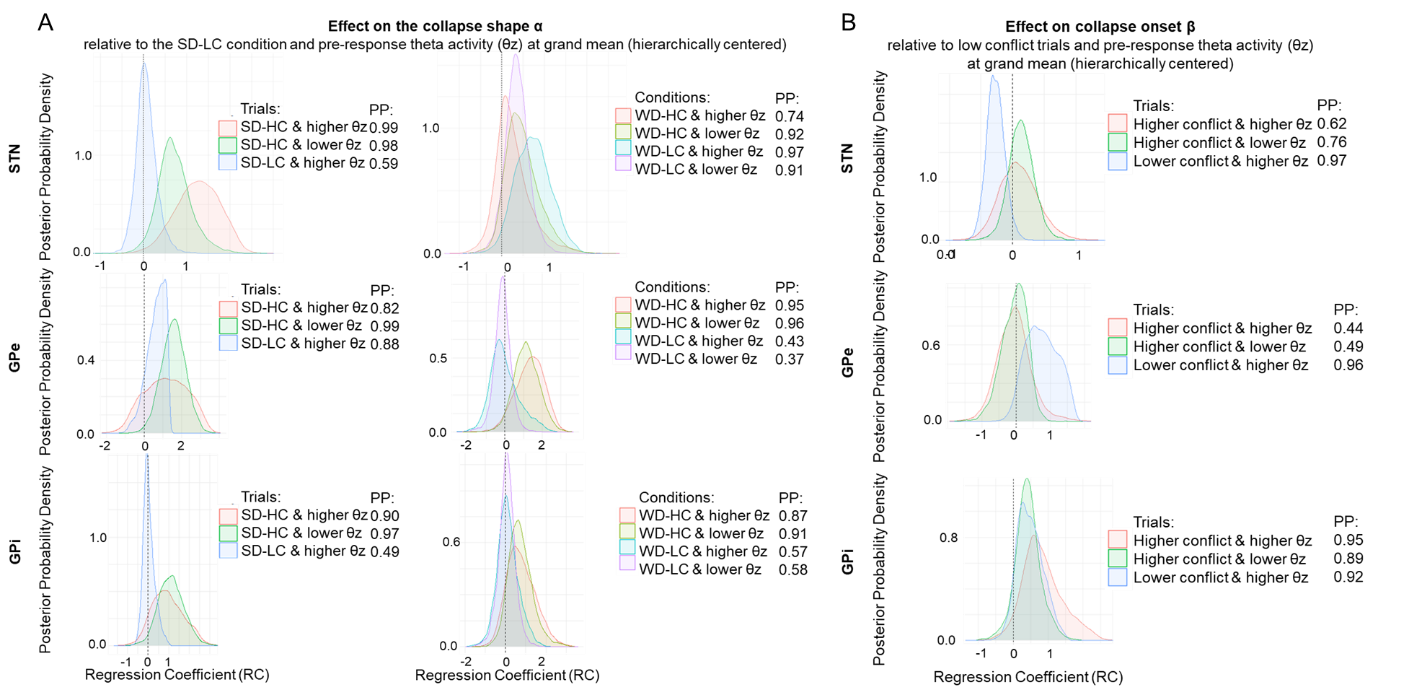


S12 Fig. Posterior estimates of best-fitting (behavioral) Weibull-DDM for each BG component.

**(A)** Conflict-by-coherence interaction on collapse shape. Shown are differences in posterior distribution of collapse shapes (α) of conditions relative to the easiest SD-LC condition for pre-response theta activity at the grand mean level. PP=Posterior probability (PP) for systematic difference between regression coefficients (relative to the easiest SD-LC condition and for grand mean pre-response theta activity. **(B)** Main effect of high-low conflict on the onset of boundary collapse. Shown is the difference in posterior distribution of collapse onsets (β) between higher versus lower conflict trials. PP=Posterior probability (PP) for systematic difference between regression coefficients (relative to low conflict trials and for grand mean pre-response theta activity. Report on conflict-induced effects (comparing low versus high conflict for a given theta level): Under stronger discriminability, higher conflict was associated with less boundary collapse as compared to lower conflict (for theta activity at the grand average: θ_z|mean_). This conflict-induced response cautiousness due to more concave collapse shape was observed in all three BG components (PP for STN: α_SD-LC,θz|mean_ > α_SD-HC,θz|mean_ = 0.9665; PP for GPe: α_SD-LC,θz|mean_ > α_SD-HC,θz|mean_ = 0.9915; PP for GPi: α_SD-LC,θz|mean_ > α_SD-HC,θz|mean_ = 0.9690). Under weaker discriminability, higher conflict was associated with less boundary collapse as compared to lower conflict but only for the GPe and GPi (PP for STN: α_WD-LC,θz|mean_ > α_WD-HC,θz|mean_ = 0.8188; PP for GPe: α_WD-LC,θz|mean_ > α_WD-HC,θz|mean_ = 0.9800; PP for GPi: α_WD-LC,θz|mean_ > α_WD-HC,θz|mean_ = 0.8190). Report additional theta-modulated effect for higher conflict trials: Under stronger discriminability, higher theta activation in the STN additionally promoted response cautiousness by prolongating boundary collapse (PP for STN: α_SD-HC,θz|higher_ > α_SD-HC,θz|mean_ = 0.9296; PP for GPe: α_SD-HC,θz|higher_ > α_SD-HC,θz|mean_ = 0.3319; PP for GPi: α_SD-HC,θz|higher_ > α_SD-HC,θz|mean_ = 0.3527). Under weaker discriminability though, higher theta activation in the GPe additionally promoted response cautiousness by prolongating boundary collapse (PP: α_WD-HC,θz|higher_ > α_WD-LC,θz|mean_ = 0.9708). This effect was similar for higher conflict trials with easier discriminability (PP: α_SD-HC,θz|higher_ > α_SD-LC,θz|mean_ = 0.82). Moreover, these effects were specific to GPe, we did neither find them in the STN nor in the GPi (PP for STN: α_WD-LC,θz|mean_ > α_WD-HC,θz|mean_ = 0.8391; PP for GPi: α_WD-LC,θz|mean_ > α_WD-HC,θz|mean_ = 0.5219). Report different theta-related modulation for stronger versus weaker discriminability: Importantly, higher conflict induced distinct theta-moderated decision dynamics under weaker than stronger discriminability in the STN. For higher conflict under weaker discriminability, higher pre-response theta activation in the STN was associated with less concave collapse shape (PP for STN: α_SD-HC,θz|higher_ > α_WD-HC,θz|higher_ = 0.9498) facilitating faster responding. These discriminability-dependent conflict differences were specific to the STN (PP for GPe: α_SD-HC,θz|higher_ > α_WD-HC,θz|higher_ = 0.5525; PP for GPi: α_SD-HC,θz|higher_ > α_WD-HC,θz|higher_ = 0.4260). We provide data and scripts on:

<https://osf.io/k38pj/?view_only=5c442294fcfb4991bb42cd902c60249c>
