# Supplementary material for: Basal ganglia components have distinct computational roles in decision-making dynamics under conflict and uncertainty
Source: PLoS Biol. 2025 Jan 23;23(1):e3002978. doi: 10.1371/journal.pbio.3002978 (PMC11756759; doi:10.1371/journal.pbio.3002978)
Supplement: S13 Fig — (DOCX) [file pbio.3002978.s014.docx]

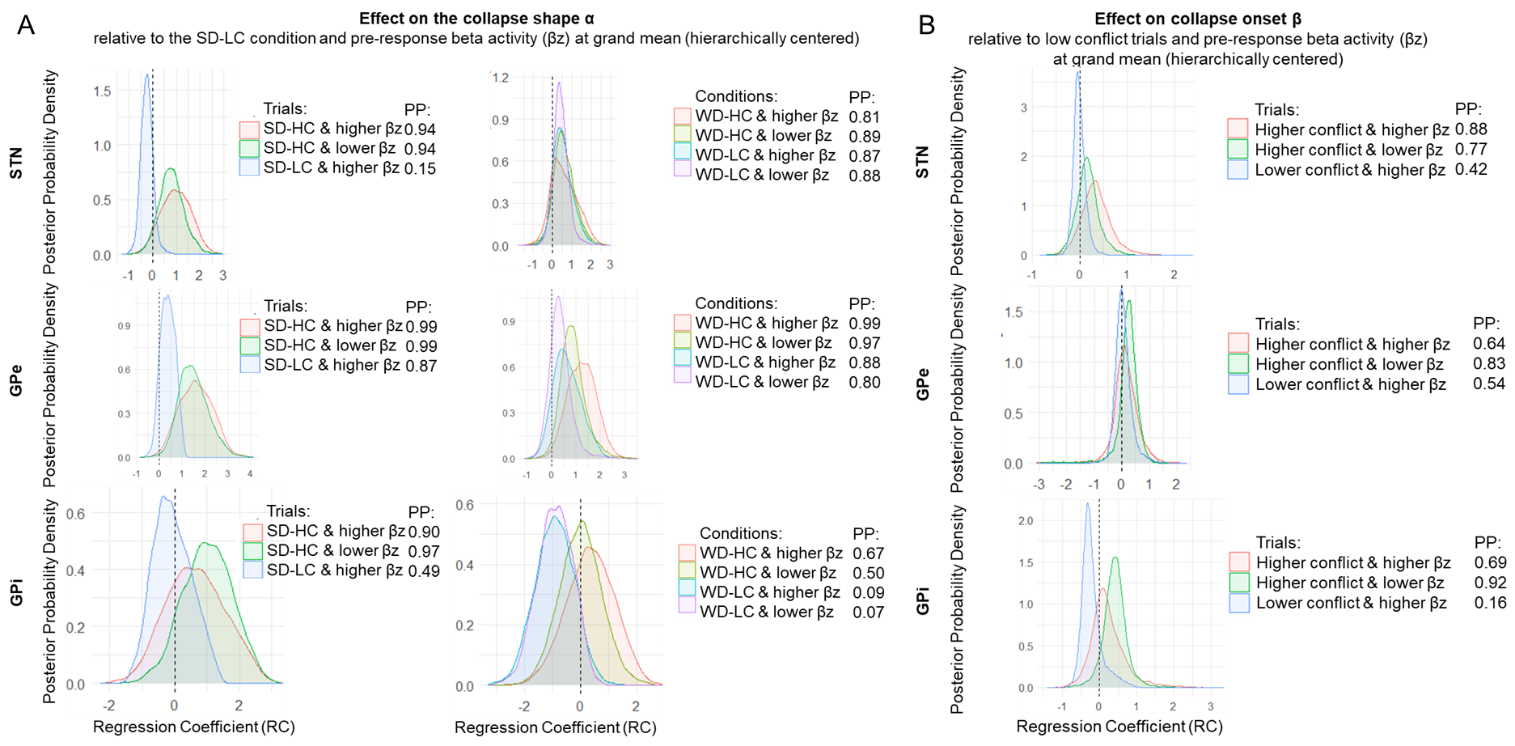
S13 Fig. Posterior estimates of best-fitting (behavioral) Weibull-DDM with beta frequency band activity as covariate for each BG component.

**(A)** Conflict-by-coherence interaction on collapse shape. Shown are differences in posterior distribution of collapse shapes (α) of conditions relative to the easiest SD-LC condition for pre-response beta activity at the grand mean level. PP=Posterior probability (PP) for systematic difference between regression coefficients (relative to the easiest SD-LC condition and for grand mean pre-response beta activity. **(B)** Main effect of high-low conflict on the onset of boundary collapse. Shown is the difference in posterior distribution of collapse onsets (β) between higher versus lower conflict trials. PP=Posterior probability (PP) for systematic difference between regression coefficients (relative to low conflict trials and for grand mean pre-response beta activity. Unlike theta (see Supplementary Figure 12), higher beta did not affect decision boundary dynamics under higher conflict and stronger discriminability in any subregion (PP for STN: α_SD-HC,βz|higher_ > α_SD-HC,βz|mean_ = 0.7100; PP for GPe: α_SD-HC,βz|higher_ > α_SD-HC,βz|mean_ = 0.5611; PP for GPi: α_SD-HC,βz|higher_ > α_SD-HC,βz|mean_ = 0.4334). Unlike theta (see Supplementary Figure 12), higher beta in the GPe did not affect decision boundary dynamics under higher conflict and weaker discriminability (PP: α_WD-HC,βz|higher_ > α_WD-LC,βz|mean_ = 0.8174). Unlike theta (see Supplementary Figure 12), higher beta in the STN did not affect decision boundary dynamics under higher conflict and weaker discriminability (PP for STN: α_SD-HC,βz|higher_ > α_WD-HC,βz|higher_ = 0.6200). We provide data and scripts on:

<https://osf.io/k38pj/?view_only=5c442294fcfb4991bb42cd902c60249c>
