# Supplementary material for: Basal ganglia components have distinct computational roles in decision-making dynamics under conflict and uncertainty
Source: PLoS Biol. 2025 Jan 23;23(1):e3002978. doi: 10.1371/journal.pbio.3002978 (PMC11756759; doi:10.1371/journal.pbio.3002978)
Supplement: S14 Fig — (DOCX) [file pbio.3002978.s015.docx]

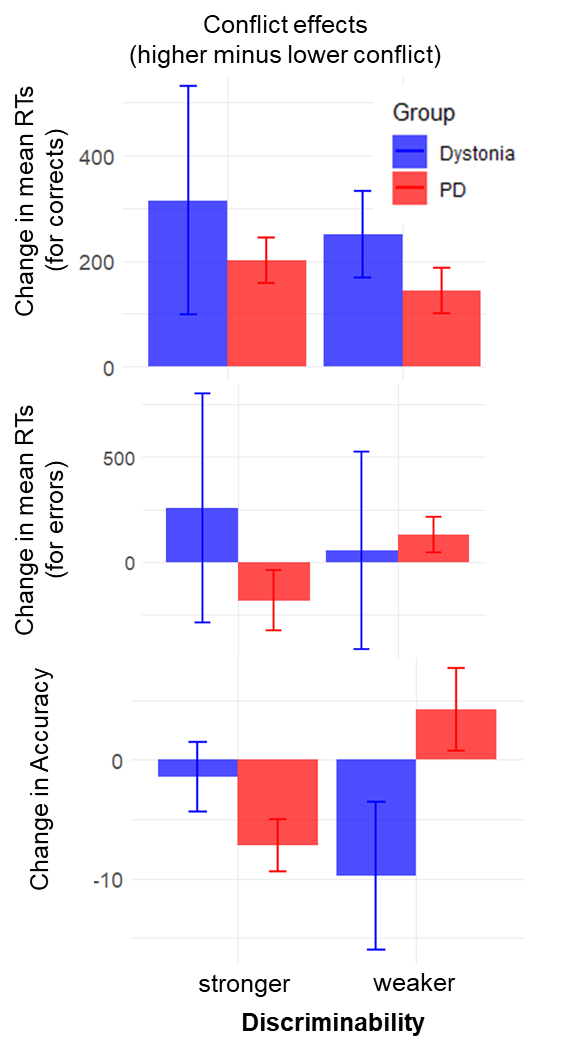


S14 Fig. Differences in conflict effects by Parkinson’s Disease (PD) versus Dystonia.

Changes in mean reaction times for correct responses (top row) and error responses (middle row) as well as accuracy (bottom row) for high minus low conflict trials for each discriminability level (stronger versus weaker). All measures were calculated by subject and then averaged across subjects. Vertical bars represent standard errors in means. Our participant pool included 4 patients diagnosed with Dystonia and 11 patients diagnosed with Parkinson’s Disease (PD). All recordings from those diagnosed with Dystonia were either in the GPe or GPi while patients with PD had recordings from either the STN, GPe, and/or GPi. Both patient groups show a slow-down of correct responses in response to conflict (irrespective of discriminability level). We provide data and corresponding analyses scripts for reproducing figures on:

<https://osf.io/k38pj/?view_only=5c442294fcfb4991bb42cd902c60249c>
