# Supplementary material for: Basal ganglia components have distinct computational roles in decision-making dynamics under conflict and uncertainty
Source: PLoS Biol. 2025 Jan 23;23(1):e3002978. doi: 10.1371/journal.pbio.3002978 (PMC11756759; doi:10.1371/journal.pbio.3002978)
Supplement: S15 Fig — (DOCX) [file pbio.3002978.s016.docx]

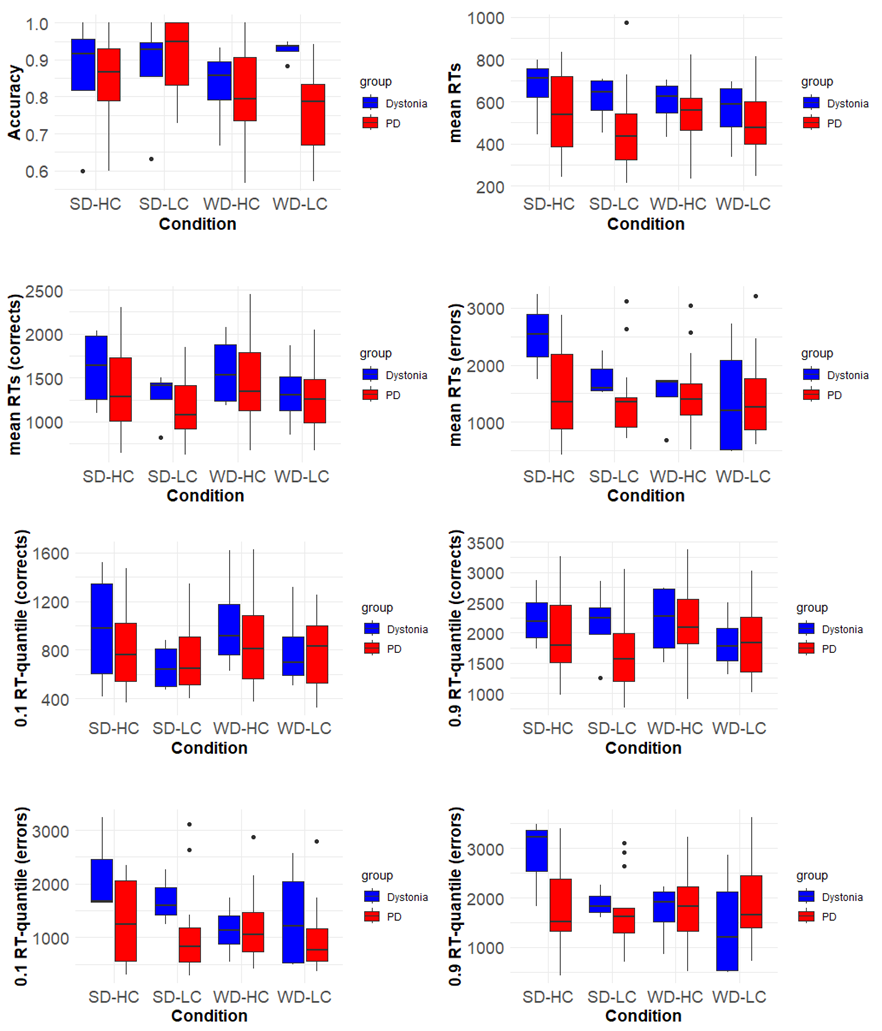


S15 Fig. Behavioral performance by Parkinson’s Disease (PD) versus Dystonia.

Box plots for accuracy, mean reaction times (averaged across correct and error responses) as well as mean reaction times, 0.1 RT quantiles (fastest responses) and 0.9 RT quantiles (slowest responses) for corrects and errors, respectively. All measures were calculated by subject and then averaged across subjects. Vertical bars represent standard errors in means. Our participant pool included 4 patients diagnosed with Dystonia and 11 patients diagnosed with Parkinson’s Disease (PD). All recordings from those diagnosed with Dystonia were either in the GPe or GPi while patients with PD had recordings from either the STN, GPe, and/or GPi. We provide data and corresponding analyses scripts for reproducing figures on:

<https://osf.io/k38pj/?view_only=5c442294fcfb4991bb42cd902c60249c>
