# Supplementary material for: Basal ganglia components have distinct computational roles in decision-making dynamics under conflict and uncertainty
Source: PLoS Biol. 2025 Jan 23;23(1):e3002978. doi: 10.1371/journal.pbio.3002978 (PMC11756759; doi:10.1371/journal.pbio.3002978)
Supplement: S16 Fig — (DOCX) [file pbio.3002978.s017.docx]

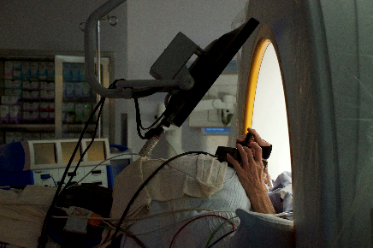


S16 Fig. Illustration of recording setup.

A patient performing an intraoperative task using a joystick. It illustrates the patient's positioning within an intraoperative CT scanner, offering a practical context to our study's methodology.
