# Supplementary material for: Basal ganglia components have distinct computational roles in decision-making dynamics under conflict and uncertainty
Source: PLoS Biol. 2025 Jan 23;23(1):e3002978. doi: 10.1371/journal.pbio.3002978 (PMC11756759; doi:10.1371/journal.pbio.3002978)
Supplement: S17 Fig — (DOCX) [file pbio.3002978.s018.docx]

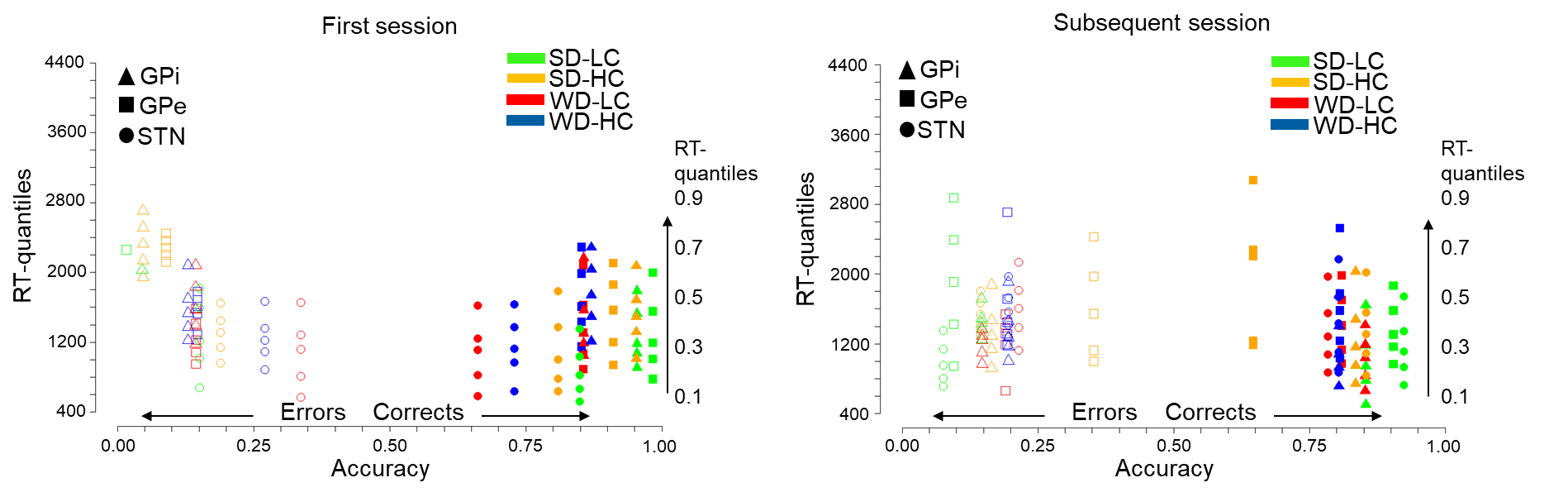


S17 Fig. Quantile-probability plots by recording sessions.

Quantile-probability plots display response frequency along the x-axis and RT quantiles along the y-axis (i.e., the .1, .3, .5, .7, and .9 RT quantile from bottom to top, respectively). Shown are the behavioral patterns for subjects’ first session (left subplot) by their recording site (GPi, GPe, and STN), and for their subsequent session (right subplot). Supplemental Table I shows that most participants had only one additional session. Task conditions: SD-LC = stronger discriminability, lower conflict; SD-HC = stronger discriminability, higher conflict; WD-LC = weaker discriminability, lower conflict; WD-HC = weaker discriminability, higher conflict. We provide data and corresponding analyses scripts for reproducing figures on:

<https://osf.io/k38pj/?view_only=5c442294fcfb4991bb42cd902c60249c>
