# Supplementary material for: Basal ganglia components have distinct computational roles in decision-making dynamics under conflict and uncertainty
Source: PLoS Biol. 2025 Jan 23;23(1):e3002978. doi: 10.1371/journal.pbio.3002978 (PMC11756759; doi:10.1371/journal.pbio.3002978)
Supplement: S18 Fig — (DOCX) [file pbio.3002978.s019.docx]

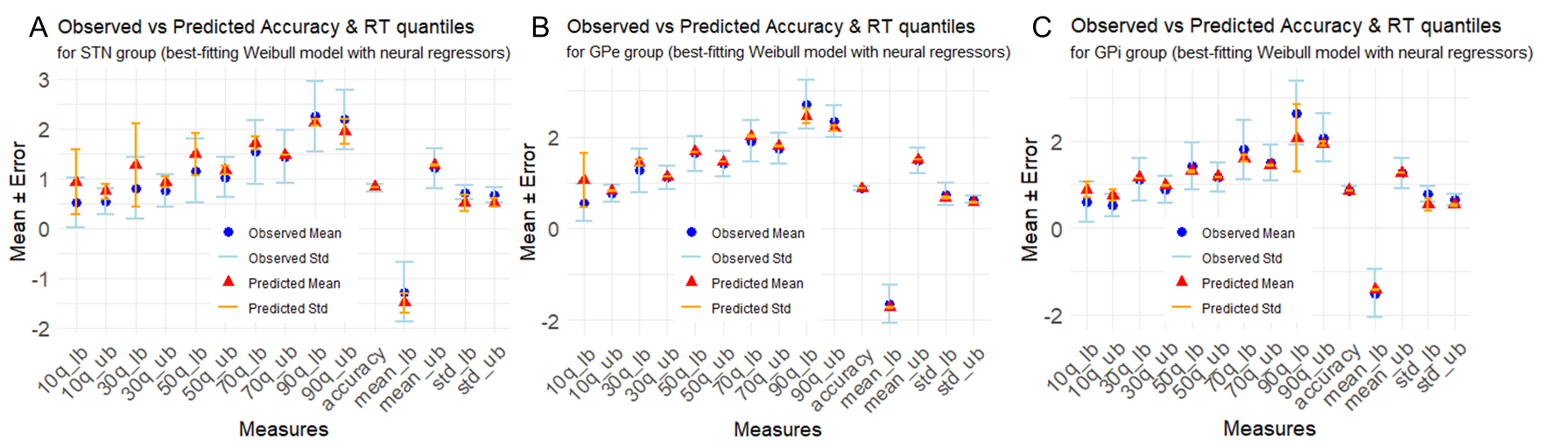
S18 Fig. Posterior predictive checks for best-fitting Weibull model with neural regressors.

The figure compares observed and predicted accuracy and reaction time (RT) quantiles. The quantiles (10q, 30q, 50q, 70q, 90q) represent the 0.1, 0.3, 0.5, 0.7, and 0.9 RT distributions, respectively. Error responses, denoted as “lb” (lower boundary), reflect decisions terminating at the lower decision boundary of the best-fitting Weibull model, while correct responses, denoted as “ub” (upper boundary), reflect decisions terminating at the upper decision boundary. **A**. posterior predictive check for the STN group. **B**. posterior predictive check for the GPe group. **C**. posterior predictive check for the GPi group. We provide data and corresponding analyses scripts for reproducing figures on:

<https://osf.io/k38pj/?view_only=5c442294fcfb4991bb42cd902c60249c>
