# Supplementary material for: Basal ganglia components have distinct computational roles in decision-making dynamics under conflict and uncertainty
Source: PLoS Biol. 2025 Jan 23;23(1):e3002978. doi: 10.1371/journal.pbio.3002978 (PMC11756759; doi:10.1371/journal.pbio.3002978)
Supplement: S1 Table — (DOCX) [file pbio.3002978.s020.docx]

Supplementary Table 1 Characteristics of patients with intracranial recordings.

| **ID** | **subjectID** | **Region** | **Diagnose** | **Brain** | **Handedness** | **Nr of trials** | **Position** |
| --- | --- | --- | --- | --- | --- | --- | --- |
| 1 | s02TT_B | STN | PD | Left | Right | 80 | Anterior-Center-Posterior |
| 2 | s02TT_C | STN | PD | Left | Right | 79 | Anterior-Center-Posterior |
| 3 | s02TT_E | STN | PD | Right | Right | 90 | Anterior-Center-Posterior |
| 4 | s05BS_B | STN | PD | Left | Right (but Left used) | 77 | Anterior-Center-Posterior |
| 5 | s05BS_C | STN | PD | Left | Right (but Left used) | 80 | Anterior-Center-Posterior |
| 6 | s05BS_D | STN | PD | Right | Right (but Left used) | 100 | Anterior-Center-Posterior |
| 7 | s06HW_B | STN | PD | Left | Right | 74 | Anterior-Center-Posterior |
| 8 | s06HW_C | STN | PD | Right | Right | 76 | Anterior-Center-Posterior |
| 9 | s15DE_A | STN | PD | Left | Right | 90 | Anterior-Center-Posterior |
| 10 | s26BV_A | STN | PD | Both | Right | 120 | Anterior-Center-Posterior |
| 11 | s26BV_B | STN | PD | Both | Right | 120 | Anterior-Center-Posterior |
| 12 | s23AT_A | STN | PD | Both | Right | 120 | Anterior-Center-Posterior-Lateral |
| 13 | s0117KN_B | STN | PD | Left | Right | 60 | NA |
| 14 | s0123AR_A | STN | PD | Left | Right | 132 | NA |
| 15 | s0123AR_B | STN | PD | Left | Right | 147 | NA |
| 16 | s34XH_A | GPe | Dystonia | Left | Left | 73 | Anterior-Middle-Lateral |
| 17 | s34XH_F | GPi | Dystonia | Left | Left | 80 | Anterior-Middle-Lateral |
| 18 | s01KO_A | GPe | Dystonia | Left | Right | 81 | Anterior-Middle-Lateral |
| 19 | s01KO_B | GPe | Dystonia | Left | Right | 100 | Anterior-Middle-Lateral |
| 20 | s01KO_C | GPi | Dystonia | Left | Right | 83 | Anterior-Middle-Lateral |
| 21 | s20EH_A | GPe | Dystonia | Both | Right | 81 | Anterior-Center-Posterior |
| 22 | s20EH_B | GPi | Dystonia | Both | Right | 84 | Anterior-Center-Posterior |
| 23 | s38KG_A | GPi | PD | Both | Right | 120 | Anterior-Center-Posterior |
| 24 | s61WM_A | GPe | PD | Both | Right | 92 | Anterior-Center-Posterior |
| 25 | s61WM_B | GPi | PD | Both | Right | 92 | Anterior-Center-Posterior |
| 26 | s74ZJ_A | GPi | PD | Left | Right | 121 | Anterior-Center-Posterior |

Abbreviations: STN = subthalamic nucleus; GPe = globus pallidus externus; GPi = globus pallidus internus; PD = Parkinson’s disease; NA = missing information. ID refers to the subject’s identifier to match the behavioral data. subjectID refers to the subject’s identifier to match neuronal data.

We provide data and scripts on:

<https://osf.io/k38pj/?view_only=5c442294fcfb4991bb42cd902c60249c>
