# Supplementary material for: Basal ganglia components have distinct computational roles in decision-making dynamics under conflict and uncertainty
Source: PLoS Biol. 2025 Jan 23;23(1):e3002978. doi: 10.1371/journal.pbio.3002978 (PMC11756759; doi:10.1371/journal.pbio.3002978)
Supplement: S2 Table — (DOCX) [file pbio.3002978.s021.docx]

Supplementary Table 2 Comparison of sequential sampling models for patient group.

| **Label** | **Version** | **DIC** | **Drift rate (v)** | **Boundary separation (a)** | **Non-decision Time (Ter)** | **Model version-specific parameters** |
| --- | --- | --- | --- | --- | --- | --- |
| Best | weibull | 5035 | 1 + coherence | 1 | 1 | alpha ~ 1 + coherence*conflict; beta ~ 1 + conflict |
| 2 | weibull | 5042 | 1 + coherence | 1 | 1 | alpha ~ 1 + coherence*conflict; beta ~ 1 |
| 3 | weibull | 5048 | 1 + coherence | 1 | 1 | alpha ~ 1 + conflict; beta ~ 1 + conflict*coherence |
| *4* | *weibull* | *5050* | *1 + coherence* | *1* | *1* | *alpha ~ 1 + coherence*conflict; beta ~ 1 + conflict*coherence* |
| 5 | angle | 5049 | 1 + coherence | 1 | 1 | angle ~ 1 + conflict |
| 6 | angle | 5049 | 1 + coherence | 1 | 1 | angle ~ 1 + coherence*conflict |
| 7 | angle | 5052 | 1 + coherence | 1 + conflict | 1 | angle ~ 1 |
| 8 | angle | 5052 | 1 + coherence | 1 + conflict | 1 | angle ~ 1 + conflict |
| 9 | angle | 5054 | 1 + coherence | 1 + coherence*conflict | 1 | angle ~ 1 |
| 10 | ou | 5260 | 1 + coherence | 1 | 1 | g ~ 1 + conflict |
| 11 | ou | 5262 | 1 + coherence | 1 + conflict | 1 | g ~ 1 |
| *12* | *ou* | *5263* | *1 + coherence* | *1* | *1* | *g ~ 1 + conflict*coherence* |
| 13 | ou | 5265 | 1 + coherence | 1 + conflict*coherence | 1 | g ~ 1 |
| 14 | ddm | 5235 | 1 + coherence | 1 + conflict | 1 | st ~ 1; η ~1 |
| 15 | ddm | 5339 | 1 + coherence | 1 + conflict | 1 | η ~1 |
| 16 | ddm | 5343 | 1 + coherence | 1 + conflict | 1 | no variability measures |
| 17 | ddm | 5347 | 1 + coherence | 1 + conflict*coherence | 1 | no variability measures |

Results from model comparison for the behavioral patterns of the patient groups (i.e., participants with intracranial recordings in the basal ganglia). Each row represents a different sequential sampling model with the best-fitting model represented on top. The variables are described in the Methods. Models in italics did not converge. angle = model with a linearly collapsing boundary (angle) indexing the angle. ddm = diffusion decision model with st referring to variability in nondecision time; η referring to across-trial variability in drift rate. weibull = model with Weibull-informed collapsing boundaries with parameters α indicating collapse shape and β indicating collapse onset. ou = Ornstein-uhlenbeck model with g indicating the decay parameter of diffusion processes. 1 refers to intercepts. DIC refers to the deviance information criterion.
