# Supplementary material for: Basal ganglia components have distinct computational roles in decision-making dynamics under conflict and uncertainty
Source: PLoS Biol. 2025 Jan 23;23(1):e3002978. doi: 10.1371/journal.pbio.3002978 (PMC11756759; doi:10.1371/journal.pbio.3002978)
Supplement: S3 Table — (DOCX) [file pbio.3002978.s022.docx]

Supplementary Table 3 Comparison of sequential sampling models for student (control) group.

| **Label** | **Version** | **DIC** | **Drift rate (v)** | **Boundary separation (a)** | **Non-decision Time (Ter)** | **Model version-specific parameters** |
| --- | --- | --- | --- | --- | --- | --- |
| Best | weibull | 29471 | 1 + coherence | 1 | 1 | alpha ~ 1 + coherence*conflict; beta ~ 1 + conflict |
| 1 | weibull | 29641 | 1 + coherence | 1 | 1 | alpha ~ 1 + coherence*conflict; beta ~ 1 |
| 2 | angle | 29656 | 1 + coherence | 1 + conflict | 1 | angle ~ 1 |
| 3 | angle | 29826 | 1 + coherence | 1 | 1 | angle ~ 1 + conflict |
| 4 | ou | 31508 | 1 + coherence | 1 | 1 | g ~ 1 + conflict |
| 5 | ou | 31510 | 1 + coherence | 1 + conflict | 1 | g ~ 1 |
| 6 | ddm | 30963 | 1 + coherence | 1 + conflict | 1 | st ~ 1; η ~1 |
| 7 | ddm | 32376 | 1 + coherence | 1 + conflict | 1 | η ~1 |

Results from model comparison for the behavioral patterns of the student group without any neurological disorders. Each row represents a different sequential sampling model with the best-fitting model represented on top. The variables are described in the Methods. Models in italics did not converge. angle = model with a linearly collapsing boundary (angle) indexing the angle. ddm = diffusion decision model with st referring to variability in nondecision time; η referring to across-trial variability in drift rate. weibull = model with Weibull-informed collapsing boundaries with parameters α indicating collapse shape and β indicating collapse onset. ou = Ornstein-uhlenbeck model with g indicating the decay parameter of diffusion processes. 1 refers to intercepts. DIC refers to the deviance information criterion.
