# Supplementary material for: Basal ganglia components have distinct computational roles in decision-making dynamics under conflict and uncertainty
Source: PLoS Biol. 2025 Jan 23;23(1):e3002978. doi: 10.1371/journal.pbio.3002978 (PMC11756759; doi:10.1371/journal.pbio.3002978)
Supplement: S4 Table — (DOCX) [file pbio.3002978.s023.docx]

Supplementary Table 4 Posterior distribution of group parameters of best-fitting model.

|  |  | STN group (n=15) | | | GPe group (n=5) | | | GPi group (n=6) | | |
| --- | --- | --- | --- | --- | --- | --- | --- | --- | --- | --- |
| **parameter** | **coefficient** | **mean** | **lower** | **upper** | **mean** | **lower** | **upper** | **mean** | **lower** | **upper** |
| boundary separation (a) | intercept | 1.508 | 0.832 | 2.240 | 1.877 | 1.095 | 2.462 | 1.726 | 0.860 | 2.426 |
| nondecision time (Ter) | intercept | 0.233 | 0.028 | 0.376 | 0.233 | 0.010 | 0.620 | 0.183 | 0.010 | 0.432 |
| drift rate (v) | intercept | 1.022 | 0.084 | 1.077 | 0.991 | 0.626 | 1.506 | 1.225 | 0.633 | 2.174 |
|  | discriminability | -0.380 | -0.517 | -0.247 | -0.170 | -0.521 | 0.177 | -0.250 | -0.462 | -0.031 |
| collapse shape (α) | intercept | 1.423 | 0.477 | 2.267 | 1.657 | 0.452 | 3.315 | 1.359 | 0.447 | 2.539 |
|  | discriminability | 0.354 | -0.176 | 0.942 | 0.091 | -1.335 | 1.472 | 0.066 | -0.786 | 0.908 |
|  | conflict | 0.723 | 0.012 | 1.628 | 1.368 | -0.252 | 2.737 | 1.119 | -0.070 | 2.395 |
|  | discriminability:conflict | -0.590 | -1.622 | 0.443 | -0.478 | -2.250 | 1.403 | -0.451 | -1.942 | 1.197 |
|  | zθ | 0.065 | -0.366 | 0.597 | 0.468 | -0.969 | 1.663 | 0.041 | -0.417 | 0.709 |
|  | discriminability:zθ | 0.313 | -0.360 | 0.998 | -0.374 | -2.146 | 1.646 | 0.006 | -0.828 | 0.775 |
|  | conflict:zθ | 0.471 | -0.406 | 1.186 | -1.072 | -3.324 | 1.104 | -0.201 | -1.220 | 0.908 |
|  | discriminability:conflict:zθ | -1.071 | -2.119 | 0.048 | 0.899 | -1.913 | 3.652 | 0.192 | -1.231 | 1.549 |
| collapse onset (β) | intercept | 2.374 | 1.990 | 2.825 | 2.376 | 1.302 | 3.368 | 2.144 | 0.819 | 3.226 |
|  | conflict | 0.138 | -0.269 | 0.563 | 0.126 | -0.802 | 1.119 | 0.421 | -0.343 | 1.223 |
|  | zθ | -0.268 | -0.536 | 0.026 | 0.830 | -0.384 | 2.080 | 0.482 | -0.180 | 1.265 |
|  | conflict:zθ | 0.232 | -0.230 | 0.742 | -0.583 | -1.970 | 1.060 | -0.071 | -1.163 | 0.819 |

The terms “lower” and “upper” refer to the respective lower and upper bounds of the 95% highest density interval of the posterior distribution. The best-fitting model is specified in the Method section. We follow standard notation of regression-based models. STN = patients with intracranial recordings in the subthalamic nucleus. GPe = patients with intracranial recordings in the globus pallidus externus. GPi = patients with intracranial recordings in the globus pallidus internus. We provide data and scripts on:

<https://osf.io/k38pj/?view_only=5c442294fcfb4991bb42cd902c60249c>
